# Updating the *Status quo* on the Eco-Friendly Approach for Antioxidants Recovered from Plant Matrices Using Cloud Point Extraction

**DOI:** 10.3390/antiox13030280

**Published:** 2024-02-25

**Authors:** Vanja Travičić, Teodora Cvanić, Olja Šovljanski, Tamara Erceg, Milica Perović, Alena Stupar, Gordana Ćetković

**Affiliations:** 1 Faculty of Technology Novi Sad, University of Novi Sad, Bulevar cara Lazara 1, 21000 Novi Sad, Serbia; vanjaseregelj@tf.uns.ac.rs (V.T.); teodora.cvanic@uns.ac.rs (T.C.); oljasovljanski@uns.ac.rs (O.Š.); tamara.erceg@uns.ac.rs (T.E.); perovicmilica@uns.ac.rs (M.P.); 2 Institute of Food Technology, University of Novi Sad, Bulevar cara Lazara 1, 21000 Novi Sad, Serbia; alena.tomsik@fins.uns.ac.rs

**Keywords:** cloud point extraction, micelle extraction, antioxidants, bioactive compouns, surfactants, micelles, recovery, green chemistry, eco-friendly extraction

## Abstract

The concepts of “green chemistry” are gaining importance in the agri-food sector due to the need to minimize pollution from toxic chemicals, improve the safety and sustainability of industrial processes, and provide “clean-labeled products” required by consumers. The application of the cloud point extraction (CPE) is considered a promising alternative to conventional organic solvents. In the CPE, the separation of compounds from the bulk solution occurs by adding a surfactant (either non-ionic or ionic). When the solution is heated to or above a critical temperature, referred to as the cloud point, two phases are formed—micellar and aqueous. Recently, the horizons of the traditional CPE have been increasingly expanding by improved procedures and integration with other techniques, such as the microwave- and ultrasonic-assisted extraction. This article provides an updated overview of the theory and research articles on the CPE from 2018 to 2023 and critically discusses the issues relevant to the potential applicability of the CPE as a promising and green technique for antioxidants recovered from plant materials. Finally, some future perspectives and research needs for improved CPE are presented.

## 1. Introduction

### 1.1. Rationale behind the Study

In recent years, there has been a growing interest in bioactive compounds such as pigments, minerals, polysaccharides, organic acids, dietary fibers, sugars, lipids, and phytochemicals (polyphenols, carotenoids) that not only serve as nutrients but also provide therapeutic outcomes to consumers [1]. Advances in the strategy of their extraction from plants have mostly been encouraged by the 12 principles of green chemistry; three of them concern the use of solvents, safer reaction conditions, and waste prevention. The toxic and volatile nature of many organic solvents commonly used in the recovery of plant antioxidants raised concerns about the environment and human health and launched innovations in extraction techniques [2,3].

Currently, conventional techniques, including maceration, percolation, decoction, solid–liquid extraction, or liquid–liquid extraction, have been employed to extract these antioxidants [2]. Some of these techniques involve the use of chemical solvents, which are widely recognized to be of great environmental concern. In addition, it requires long extraction time, more energy consumption, challenges in the removal of solvents, additional purification steps, lower extraction efficiency, safety, poor extract quality, and high total cost [3]. Alternative extraction techniques like pressurized water extraction, enzyme-assisted extraction, supercritical fluid extraction, microwave extraction, ultrasound extraction, and solid–liquid partitioning are considered more environmentally friendly, but they require specialized equipment to operate and need skills and additional costs, and they are energy-consuming [2].

### 1.2. Cloud Point Extraction (CPE)

Cloud point extraction (CPE), also known as the surfactant-based extraction, liquid-concentration technique, micelle extraction, or micelle-mediated extraction [4], is an innovative and eco-friendly approach for antioxidant recovery, particularly from food. Scientists have been exploring its potential for the past 20 years and adding new uses [5]. As a result, numerous studies have been published that cover its theoretical background, especially proposing the CPE technique for the preconcentration of trace elements before analysis related to toxicity, pollution, and food quality evaluations. However, research on the use of the CPE for the extraction of valuable organic compounds is still relatively rare [6,7,8,9].

The CPE technique is predicated on the use of surfactants to separate compounds from the bulk solution. This process leads to the formation of clouds when the solution is heated to or above a critical temperature, commonly referred to as a cloud point [10]. Surfactants applied in the CPE can be either non-ionic or ionic. The separation of the desired compound from the bulk solution can also be enhanced by adding salt (salting-out phenomenon) [11,12]. Surfactants are a group of amphiphilic molecules that contain a hydrophilic part directed toward the hydrophilic (water) phase and a hydrophobic part directed toward the hydrophobic (oil) layer. Depending on the type of surfactant and solution, the micelle structure can be roughly spherical or oval. Further, centrifugation is applied to separate the solution into two distinct phases. The bottom phase is called the surfactant-rich phase and contains most of the surfactant molecules and the hydrophobic compounds already present in the solution. The upper phase is the aqueous phase that contains any molecules or ions that cannot be incorporated into the micellar system. The phase separation is caused by heat-induced dehydration of the surfactant polar groups. This promotes micelle aggregation by decreasing repulsions between micelle molecules [11].

The low requirements of the CPE in reagents make it one of the most innovative techniques for antioxidant recovery. Furthermore, the CPE is usually carried out at mild or low temperatures and without the use of hazardous or toxic reagents. The extraction efficiency of target compounds by the CPE technique is influenced by many factors, such as the type and concentration of the surfactants, the pH of a sample solution, the temperature and duration of reaching equilibrium, and ionic strength [13,14,15,16,17]. Many recent scientific papers are precisely related to the monitoring of these factors on the extraction efficiency of target compounds as well as their optimization. The purpose of this review is to provide an updated overview of the theory and research articles on the CPE. The review will analyze and critically discuss the issues relevant to the potential applicability of the CPE as a promising and green technique for recovering antioxidants from plant materials to better understand opportunities and challenges for their implementation as an eco-friendly alternative to organic solvents.

## 2. Review Methodology—Current Literature Gap or Not?

**Figure 1:**
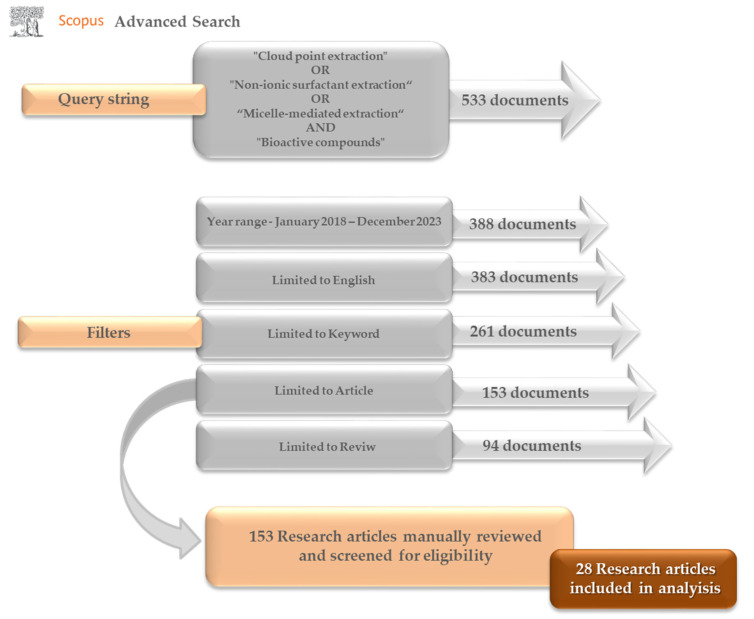
Scopus database search.

In December 2023 (assessed on 4 December 2023), the Scopus database was used to search for references using the following keyword combinations: “cloud point extraction” OR “non-ionic surfactant extraction” AND “bioactive compounds”. The study was limited to articles written in English, analyzing papers published from 2018 to 2023 to ensure their correlation with the topic matter. In addition, the study was limited to specific keywords, as shown in Figure 1. A total of 153 research articles were identified from database searches. All titles and abstracts were read and checked regarding their relevance for the review. The articles that investigated recovering antioxidants such as polyphenols, carotenoids, chlorophylls, etc., from different plant matrices, i.e., fruit, vegetables, algae, and their by-products, were chosen for consideration in this review.

**Figure 2:**
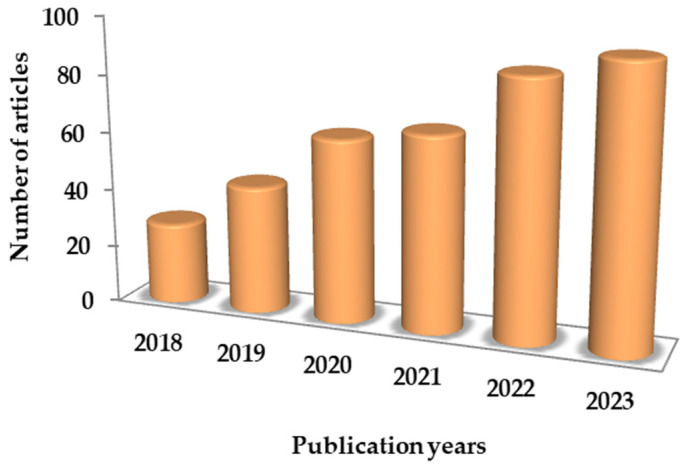
Trend in publications related to CPE extraction.

The increasing interest in the CPE approach for recovering bioactive compounds that serve as antioxidants is evident in the rising trend of publications (Figure 2). From 2018 to 2019, the development of the CPE approach was relatively slow, with 28 and 44 published articles; from 2018 to 2021 was the growth period, with an average of 65 (63 and 67, respectively) articles published. In the last two years, the CPE technique entered a fast growth period, with 88 articles in 2022 and 95 articles in 2023 (more precisely, 4 December 2023).

## 3. Experimental Protocol of CPE

**Figure 3:**
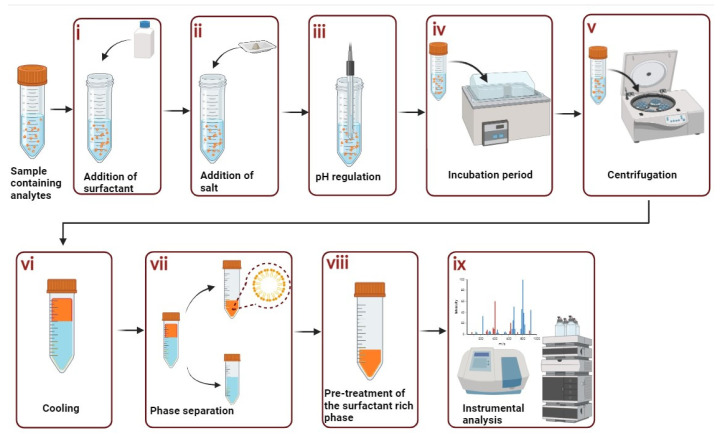
CPE protocol.

The CPE is composed of eight simple steps [13]: (i) Addition of surfactants; (ii) Addition of salt (iii) pH maintenance (iv) Incubation for clouding; (v) Centrifugation; (vi) Cooling; (vii) Phase separation for analysis; (viii) Pretreatment of the surfactant rich phase; (ix) Instrumental analysis. The scheme of the basic CPE protocol is shown in Figure 3.

Micelle formation is of great importance for the CPE technique. Considering non-ionic surfactants, micelles are formed when the solution is heated to a temperature above the cloud point of the applied surfactant. The temperature at which micelles are formed is referred to as the Krafft temperature. Further, the temperature at which a surfactant solution splits into two phases—a low surfactant concentration phase and a surfactant-rich coacervate—is known as cloud point [18]. The target bioactive concentration in the coacervate is also feasible since the surfactant-rich coacervate phase has a lesser volume than the surfactant-depleted phase [19]. Therefore, the extracted bioactives trapped in the micelles may be concentrated by simply changing the system temperature [18,19,20]. Upon exceeding a specific threshold of surfactant concentration, referred to as the critical micellar concentration (CMC), the surfactant molecules spontaneously combine into colloidal-sized clusters or micelles. These micelles contain between 60 and 100 monomers and are in equilibrium with a surfactant concentration in the solution close to the CMC. The occurrence of other forms, including vesicles, inverse micelles, microemulsions, monolayers, and bilayers, is contingent upon the surfactant’s composition and concentration, as well as the utilized solvent [21]. After dispersion, a low concentration of surfactant in an aqueous solution is generally found in either a monomeric or dimeric form. When dispersed in water, the micelles have a hydrophilic surface and hydrophobic core, which means that micelles can interact chemically or physically with either hydrophilic or lipophilic compounds to enhance their solubilities. This makes surfactants excellent vectors for bioactive compound extraction. The maximum bioactive concentration that can be incorporated into a given surfactant formulation is termed the maximum additive concentration [11]. 

Frequently, Micelle formation is encouraged through salt addition, like sodium sulfate or sodium chloride, and this phenomenon is known as the salting-out effect. The micellization process in ionic and amphoteric surfactants is affected by temperature due to changes in the hydrophobic and head-group interactions. When applied to an aqueous solution, the CMC of ionic surfactants shows a monotonically decreasing trend with increasing temperature, reaching a minimum at approximately 25 °C. However, beyond this point, when the temperature continues to rise, the CMC starts to increase as well, exhibiting a U-shaped behavior [22]. Contrarily, with increasing temperature, the CMC of non-ionic surfactants exhibits a decreasing trend. An elevation in thermal energy has the potential to weaken a solution’s bonding strength, resulting in a temperature-dependent turbidity due to the dehydration of ethylene oxide units and the micelles aggregation [23]. Micelles can have a wide range of morphologies, from virtually spherical to oval, depending on the type of surfactant used and the conditions of the solution. The mechanism through which extraction occurs is still unclear [24]. After the formation of the micelles, the hydrophilic and the lipophilic phases are separated by centrifugation. Due to the presence of hydrophobic molecules and most of the surfactant molecules in the solution, the lipophilic phase is the one that is surfactant-loaded. On the other hand, in the aqueous phase, all molecules or ions that cannot be merged into the micellar system are present. The number of surfactant molecules within a micelle is defined as the degree of aggregation. The micelle number can be affected by several factors, such as surfactant type and structure, the properties of the electrolyte, the concentration, the solvent nature, temperature, and the pH level of the solution [11].

## 4. Influencing Parameters

### 4.1. Surfactants Type and Concentration

Surfactants are chemical substances that can reduce the surface or interfacial tension between two liquids, liquids and gases, or liquids and solids. Bearing a hydrophilic part and a hydrophobic part, surfactants can adsorb onto interfaces and lower the tension of the interfaces [11]. This improves the interfacial properties of the material and makes it suitable for use in cleaning, surface functionalization, foaming, and emulsification processes [25]. As previously mentioned, surfactants have two parts: a hydrophobic tail that is affine to the hydrophobic layer and a hydrophilic head that is affine to the bulk solvent (water). Usually, the hydrophobic tail is a linear or branched hydrocarbon with six to twenty carbon atoms, and it might even have aromatic rings. The surfactant head group might be ionic or non-ionic [12]. A remarkable characteristic of surfactants is their ability to dissolve specific molecules via hydrophobic, electrostatic, or a combination of the two interactions. Another specificity includes the property that, by heating, the micellar solution becomes opaque at a narrow temperature range, referred to as the cloud-point temperature. When the temperature rises over the cloud point, the solution separates into an aqueous phase and a surfactant-rich phase [26]. Because there is not much of the surfactant-rich phase, an excessive enrichment factor can be produced. Consequently, the sensitivity of analysis is raised without the need for further sample cleaning or evaporation procedures [27]. The four categories of surfactants are mainly used in the CPE, depending on the nature of the hydrophilic group (Table 1) [12].

Namely, non-ionic surfactants are the most commonly used in the CPE procedures as an extraction medium for bioactive compounds recovering. Some advantages of non-ionic surfactants are their good solubility in water and organic phases, solubilization capacity, weak ionization, commercial availability, and ease of handling [28]. The most common surfactants employed to recover bioactives usually belong to the Triton X and Genapol series due to their low cloud points (25–40 °C) and eco-friendly properties [29]. For example, Triton X-114 has low CPT (25 °C) and high density, which facilitates the separation of two formed phases. The optimal critical micelle concentration (CMC) plays a crucial role in the method of the CPE, so only a narrow concentration range allows phase separation. The preconcentration factor is reduced when a surfactant is used excessively, which consequently lowers extraction efficiency. In contrast, insufficient analyte recovery is caused by a lower amount of surfactant [30].

### 4.2. Solution pH Level

One of the crucial aspects that significantly affects the CPE is the pH level of the medium, especially for ionizable compounds. The bioactive recovery could be improved by choosing the required pH for ionizable compounds and/or adding salt, which decreases their solubility in the aqueous phase; therefore, optimization of pH is an important approach for the CPE [31]. The deprotonated/protonated particles result in an electrically neutral charge; these particles usually have no strong response to the micellar aggregate and tend to distribute into the micellar phase of the non-ionic surfactant. Consequently, the quantitative extraction method occurs at pH levels where the analyte’s neutral state is more prevalent [32].

### 4.3. Salting-Out Effect

According to the salting-out concept, a relatively high concentration of electrolytes leads to the non-electrolytes’ lower solubility. The addition of salt to micellar solution increases the degree of micelles dehydration, which strengthens the hydrophobic contacts between them. If the surfactant concentration is excessively high, turbidity occurs, and phase separation is possible. The utilization of the salting-out phenomenon in the CPE eliminates the heating step, shortening the time needed for the separation process [11].

The addition of neutral salts, such as CaCl_2_ or NaCl, may influence the critical micelle concentration (CMC) value. When the surfactant is non-ionic, the presence of electrolytes decreases the CMC, which results in a lower separation efficiency [31]. The ionic surfactants enhance the ionic strength of a solution, which leads to expediting phase separation by increasing the water-phase density [33]. The addition of electrolytes increases the extraction efficiency for polar bioactives. It decreases the critical packing temperature (CPT) and enhances the efficacy of hydrophobic interactions between the surfactant and the analyte [34].

### 4.4. Temperature

The recommended temperature for bioactive compounds extraction should be 15–20 °C greater than the cloud point of the surfactant. The Krafft point of a surfactant refers to the temperature at which its solubility significantly increases in an aqueous solution. This phenomenon is widely recognized as the melting point of a hydrated solid surfactant. The Krafft point concept has been widely employed in ionic surfactants, while it has rarely been observed for non-ionics [34]. By increasing the solution temperature over the surfactant’s cloud point, phase separation occurs due to the dehydration of the micelles and the production of a turbid solution. Raising the equilibrium temperature results in a volume decrease in the surfactant-rich phase due to the disruption of hydrogen bonds and dehydration of the phase, which results in more efficient extraction [19]. However, very high temperatures may lower the recovery of the analyte due to the decomposition of thermolabile bioactives, such as polyphenols, carotenoids, tocopherols, vitamins, etc. Therefore, the most employed temperatures range from 40 to 60 °C [35]. 

Temperature increases up to the cloud point are followed by an increase in micellar size and a corresponding reduction in the CMC. The greatest analyte preconcentration factor is reached when the CPE procedure is performed at equilibration temperatures well above the cloud point temperature of the surfactant. However, the great challenge in this step is high temperatures, which may promote the decomposition and decreased recovery of bioactive compounds [13].

### 4.5. Centrifugation

The centrifugation time has no significant impact on micelle formation, but it advances phase separation in the same sense as the conventional separation of a precipitate from its initial aqueous environment. The effective centrifugation times for most CPE procedures are around 5–10 min [13]. The centrifugation time has an important role in phase separation after cloud formation. In general, shorter centrifugation time is recognized to be more advantageous for the CPE.

### 4.6. Recent Examples and Outputs of CPE for Recovering Antioxidants from Different Plant Sources

By summarizing recent examples in Table 2, the method’s adaptability across different plant materials and conditions is shown, emphasizing its effectiveness and sustainability. By illustrating its importance in the eco-friendly extraction of bioactive compounds for use in food, cosmetics, and pharmaceuticals, recent studies offer insight into recovering different groups of antioxidants from different plant sources. Moreover, the CPEs of target groups of antioxidants (polyphenols, polyphenols and flavonoids, carotenoids, and chlorophylls) were presented using the specific CPE conditions tailored to optimize recovery. The differentiation by antioxidant groups, as well as a wide range of plant sources, underscores the adaptability of the extraction process to various compound classes, each requiring unique conditions such as surfactant type, pH, temperature, and extraction time for efficient isolation.

In view of recent examples of the CPE for the recovery of polyphenols, De Araújo Padilha et al. [36] used camu-camu (*Myrciaria dubia* McVaugh) as a source of targeted antioxidants. Utilizing Triton X-114 at 7% *w*/*v*, this method aims to extract polyphenols at a low temperature (30 °C) and acidic pH (3.2), involving a lengthy extraction time (180 min) and high salt concentration (6% NaCl), indicating an emphasis on maximizing polyphenol recovery from this tropical fruit waste. In 2023, an interesting study was made by Sazdanić et al. [37] as well; they employed a dual-surfactant system (Brij S20 and Poloxamer 407) at 3% *w*/*v*, operating at a mild temperature (25 °C) and slightly acidic pH (4), with a short extraction time (45 min), suggesting an efficient method for extracting antioxidants with minimal thermal degradation. Zafar et al. [38] employed non-ionic surfactants Triton X-100 and Tween 20 at different concentrations with or without salt (KCl) to pre-concentrate and separate polyphenols from aqueous extracts of *Acalypha fruticose* leaves. Among all the treatments, extracts pretreated, Tween 20 at 8 mM in a higher temperature range (70–80 °C), without specifying pH, highlighting a focus on thermally assisted extraction to improve yields from this medicinal plant, with KCl (2%) enhancing the process. For the polyphenol extraction from *C. papaya* leaves, Lee et al. [39] formed aqueous micellar two-phase systems composed of different types and concentrations of a single non-ionic surfactant (i.e., Pluronic L-121, L-81, L-61, and L31) and determined their respective cloud point temperature. Utilizes Pluronic L-61 at a high concentration (10% *w*/*w*) and low temperature (40 °C) for a very short time (10 min), indicating a rapid and efficient method for extracting antioxidants, possibly sensitive compounds, from papaya leaves. Giovanoudis et al. [40] optimized the CPE method to separate polyphenols from apricot cannery waste. With Peg 8000 at 2% *w*/*v*, moderate temperature (65 °C), and slightly acidic pH (3.5), this method focuses on wastewater valorization, extracting antioxidants in the three-step CPE approach effectively in a short time (20 min) with NaCl (3%) as a phase separation enhancer. Furthermore, lecithin at 5% *w*/*v* and moderate temperature (40 °C) with a neutral to slightly acidic pH (3) for 30 min, coupled with NaCl (5%), suggests a lipid-based extraction targeting lipid-soluble antioxidants from winery waste [41]. To achieve that, the authors implemented multiple extraction steps with the best efficiency using the three CPE steps. In the case of the CPE of antioxidants from olive mill wastewater, two approaches are noted, one with a high surfactant concentration (12.5% *w*/*v*) at pH 3.5 and another with a lower surfactant concentration (3% *w*/*w*), both focusing on extracting antioxidants from this challenging matrix, indicating different optimization strategies. Namely, Karadag et al. [42] used single-factorial experiments and the optimization of these parameters by RSM. The optimal conditions were established to be a temperature of 65 °C, pH level of 4.5, sodium chloride concentration of 10% (*w*/*v*), and lecithin concentration of 15% (*w*/*v*). On the other hand, lecithin was also applied by Athanasiadis et al. [46], but the CPE protocol involved treatment for 20 min at 40 °C at a pH value of 3.5 and surfactant concentration of 3% *w*/*w*. Several studies used Tween 80 as a surfactant for the CPE. Kiai et al. [43] investigated the applicability of the CPE for recovering polyphenols from table olive processing wastewater and found that the optimal parameters were established as follows: 10% surfactant (*w*/*v*), pH of 2, 70 °C, and a 30-min equilibration period. A 5% *w*/*v* concentration of Tween 80 at a high temperature (65 °C) and slightly acidic pH (3.5) for 20 min, with NaCl (3%), reflected an optimized strategy for the recovery of antioxidants from peach processing by-products [44]. Moreover, focusing on the differences in antioxidant profiles between the maturity stages of peaches, the optimized the CPE protocol hints at a temperature-dependent approach (45 °C) at a slightly acidic pH (2.5), suggesting a nuanced extraction to maximize recovery from both unripe and ripe fruits [47]. 

The two examples focus on extracting polyphenols and flavonoids from pomegranate peel using Triton X-114. The first CPE protocol for polyphenols employed an 8.22% *w*/*v* surfactant concentration at a specific temperature (36.80 °C) and pH (4), optimized for a 30-min extraction from a solid–liquid ratio of 0.5 g/50 mL, with NaCl (4%) enhancing phase separation. More and Arya [47] also optimized the CPE parameters for flavonoids: 8.27%; *v*/*v* Triton X-114, 4.06%; *w*/*v* NaCl at 34.30 °C and pH 5.07. Another study used an 8% *w*/*v* surfactant concentration at a higher temperature (55 °C) and slightly higher pH (4.5), with a different solid–liquid ratio (1:30 *w*/*v*) and a significantly higher salt concentration (14%), indicating a tailored approach to maximize extraction efficiency under varied conditions [48].

Carotenoids, as a group of antioxidants, were the CPE targets in different studies. The CPE extraction from tomato wastewater targets carotenoids using lecithin as the surfactant, with varying concentrations (1 or 2% *w*/*v*) at a moderate temperature (45 °C) and pH (3.5). This method, which includes a high salt concentration (35.6%) for phase separation, adjusts the CPE steps based on the surfactant concentration, indicating a precise optimization to enhance carotenoid recovery [48]. For brown microalgae, Tween 20 at 0.046 mol/L is used, focusing on a low-temperature extraction (25 °C) for an extended period (140 min), with a very dilute solid–liquid ratio (0.02 mg/mL). This approach, without the use of salt, aims to preserve the integrity of sensitive compounds during extraction, highlighting a gentle method for isolating antioxidants from delicate microalgae sources [36].

The CPE extraction of chlorophylls from green microalgae (*Ulva* spp.) involved the aqueous solutions of sodium dodecyl sulfate (SDS) and tributyltetradecylphosphonium chloride [P4,4,4,14]Cl at a high concentration (250 mM), conducted at room temperature (25 °C) for a short duration (30 min) with a very low biomass concentration (0.01 g/mL). This method is designed to isolate chlorophylls efficiently, reflecting an optimization for delicate extraction conditions to preserve these sensitive pigments [51]. In another study, Leite et al. [16] developed a cost-effective and sustainable process for the extraction and concentration of chlorophylls from spinach leaves using aqueous solutions of non-ionic surfactants instead of volatile organic solvents. The commercial surfactant named C11-C13 9EO’s at 12.4 mM concentration is utilized at a slightly higher temperature (41 °C) for a quick extraction (30 min) from a relatively concentrated biomass (0.07 *w*/*w*). This approach likely aims at the effective extraction of chlorophylls and possibly other leaf-based antioxidants tailored to the characteristics of spinach.

In summary, all reviewed studies present the diverse CPE techniques for the concentration of antioxidants from various plant sources, utilizing specific surfactants, temperatures, pH levels, and salt concentrations to optimize extraction efficiency. These examples highlight the adaptability and effectiveness of the CPE across a range of biological matrices and target compounds, including polyphenols, flavonoids, carotenoids, and chlorophylls. The varied conditions demonstrate the method’s flexibility and potential for sustainable extraction of valuable antioxidants from waste streams and underutilized resources, emphasizing its role in advancing green chemistry and biorefinery approaches.

## 5. Expanding the Horizons of Cloud Point Extraction: Synergistic Integration with Microwave- and Ultrasonic-Assisted Extraction Techniques

**Figure 4:**
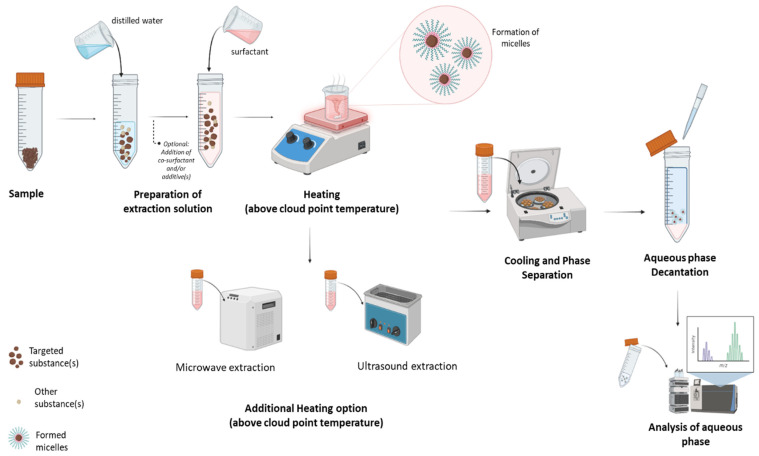
Simplified view of CPE extraction protocol with MAE or UAE.

Recent improvements in green chemistry have seen an increasing interest in the unification of the cloud point extraction (CPE) with the microwave-assisted extraction (MAE) or the ultrasonic-assisted extraction (UAE) techniques (Figure 4). For additional effectivity of the CPE, elucidating the synergistic effects with ultrasound- or microwave-supported process can be a great base for significantly enhancing the extraction efficiency and preconcentration capabilities, particularly in the context of intricate sample matrices.

### 5.1. The Cloud Point Extraction with Microwave-Assisted Extraction (CPE-MAE)

The incorporation of microwave energy into the CPE introduces a dynamic dimension to the extraction process. The interaction mechanisms, including dipolar polarization and ionic conduction, expedite the phase separation and enhance mass transfer kinetics. The CPE-MAE technique involved the surfactant-based extraction process, where a non-ionic surfactant forms micelles in the extraction solution (Figure 4). The extraction efficiency is enhanced by the application of microwave energy, which accelerates the phase separation and solubilization of analytes in the surfactant-rich phase [13,28]. The process begins with the preparation of a surfactant solution. The sample, which may be a complex matrix like biological fluids or environmental samples, needs to be prepared; for example, dissolving in a suitable solvent to ensure compatibility with the surfactant solution. Then, the surfactant solution and the sample solution are combined, and the mixture is heated to a temperature above the CPT of the surfactant. At this temperature, micelles form, encapsulating the analytes in the surfactant-rich phase while leaving the bulk of the matrix in the aqueous phase. This is the moment when microwave energy is applied to the system, introducing an additional heating mechanism. This step accelerates the extraction process by enhancing the solubilization of analytes in the micellar phase, often resulting in faster and more efficient extraction compared to traditional methods.

After microwave irradiation, the system is cooled to a temperature below the CPT. This induces phase separation, leading to the formation of two distinct phases: a surfactant-rich phase containing the solubilized analytes and an aqueous phase containing the remaining matrix components. The surfactant-rich phase, now enriched with the extracted analytes, is typically subjected to a direct analysis or back-extraction step. Back-extraction may involve the use of a suitable solvent to recover the analytes from the surfactant phase, making them amenable to further analysis, such as chromatography [28]. The explained synergistic steps between cloud point extraction and microwave-assisted extraction enhance the overall efficiency, selectivity, and speed of the extraction process, making it a valuable tool for various sample matrices. The successful implementation of the CPE-MAE relies on careful optimization of various operation parameters [13,52]. These parameters play a crucial role in determining the efficiency, selectivity, and overall performance of the integrated technique. Table 3 potted a wide-ranging list of key operation parameters that influence the CPE-MAE. Based on the presented operation parameters, a systematic categorization into distinct arrays provides a structured approach to enhancing the efficiency, selectivity, and safety of the CPE-coupled MAE extraction process. In brief, within process parameters, the CPT assumes an essential role. As previously mentioned, the precise optimization of the CPT is imperative for the CPE only, ensuring an environment conducive to the efficient phase separation required for successful analyte extraction. Concurrently, temperature control during microwave irradiation is essential to prevent the degradation of analytes, emphasizing the delicate interplay between thermodynamics and kinetics in this integrated methodology [13,52]. Stirring or agitation, a mechanical parameter within the same array, emerges as a critical contributor to mass transfer enhancement during extraction. The significance lies in its potential to improve overall extraction efficiency by optimizing the interaction between the sample and the surfactant. Moreover, the microwave power and irradiation time parameters introduce considerations of electromagnetic energy application. Their direct influence on heating and extraction efficiency necessitates careful optimization to prevent sample degradation and achieve maximal extraction yields. For further *scale-up* processes, the instrumentation parameters further extend this realm, demanding specific adjustments to the microwave instrument for harmonious compatibility with the CPE [28]. In view of the targeted sample for the CPE-coupled MAE, the importance of pretreatment cannot be overstated. The preparatory methods, such as grinding or homogenization, serve as critical interventions impacting the accessibility of analytes during extraction. Simultaneously, considerations of sample matrix characteristics and size emphasize the multifaceted nature of the sample, requiring meticulous optimization to address challenges associated with the nature, complexity, viscosity, and interference of specific samples. Regarding the role of surfactants in the CPE, the type and concentration of surfactants, intertwined with their critical micelle concentration and compatibility with microwave irradiation, demand cautious selection for effective phase separation. The incorporation of co-surfactant/additives and the optimization of the surfactant-to-sample ratio underscore additional enhancements for extraction efficiency through tailored surfactant interactions. Similarly, analyte and solvent parameters explore the nature of analytes and the choice of extraction solvents. The physicochemical properties of analytes, including solubility and volatility, introduce a layer of complexity that requires an understanding of optimal extraction conditions. Simultaneously, the choice of extraction solvent and its volume further emphasizes the necessity for particular optimization to ensure compatibility with the surfactants employed [13]. As the final tipping point for the effective CPE coupled with the MAE, some safety parameters have to be highlighted. For example, microwave vessel material emerges as a consideration directly influencing heating efficiency. While safety precautions during microwave irradiation underscore the importance of maintaining the integrity of the extraction process, critical opinions may highlight the need for standardized safety protocols to ensure reproducibility across different laboratories. To the authors’ knowledge, no safety protocols specific for this type of extraction have been established or standardized [13,52].

Since the CPE-MAE is often carried out at high pressure and high temperature but also requires extended time for cooling, filtrating, centrifuging, etc., some disadvantages can be highlighted. This extraction technique can affect the stability of samples since the application of microwave energy in the MAE may involve elevated temperatures, posing a risk to thermolabile compounds in a sample during the extraction process. The rapid and intense heating associated with the MAE may exacerbate the potential decomposition of heat-sensitive analytes. While the MAE is known for its ability to accelerate extraction processes, the overall extraction time can be extended due to steps such as cooling the sample post-microwave irradiation. This extension may be considered a drawback, especially when rapid analyses are desired. Similarly, the need for cooling in the post-MAE phase resonates with challenges associated with microwave heating. The rapid and uneven heating induced by microwaves may necessitate careful cooling to avoid thermal degradation of analytes. The following steps, such as filtration or centrifugation steps, though crucial for obtaining a clean extract, contribute to the overall processing time [28]. In the context of the MAE, these additional steps may be seen as disadvantages, especially when aiming for a streamlined and time-efficient extraction technique. Addressing these challenges requires a nuanced approach to optimize the MAE parameters and post-extraction steps, striking a balance between the benefits of accelerated extraction and the preservation of analyte integrity. One of the potential strategies to overcome the limitations of this technique is the dynamic MAE (DMAE), which represents a modification of the MAE technique. This approach enables a time-saving step by immediately transferring an extracted sample from the extraction vessel, avoiding additional degradation of analytes [53]. A similar study was conducted by Du et al. [54], where Triton X-114 was used as the micellar extraction solution, which also has a significant role in the preconcentration of targeted substances from the sample. 

The flexibility of the CPE-MAE combination is showcased through a diverse array of applications in environmental analysis, pharmaceuticals, food safety, and bioanalysis. Researchers have applied the CPE-MAE across diverse sample matrices, demonstrating its versatility in extracting targeted substances. Extraction of zinc oxide nanoparticles from environmental waters by the CPE-MAE using β-mercaptoethylamine serves to demonstrate the procedure adaptability for nanoparticle analysis in complex environmental matrices [55]. Wu et al. [53] investigated the extraction of triazine herbicides from fresh vegetables using the CPE-MAE. This application highlights the technique’s potential in selectively extracting analytes from vegetable matrices, addressing food safety and environmental monitoring challenges. In a study by Simitchiev et al. [56], the CPE-MAE was applied to extract rhodium, palladium, and platinum from trademark pharmaceutical products, illustrating the possibility of precise trace metal extraction using mercaptobenzothiazole and Triton X-100 in the pharmaceutical industry. Moreover, Du et al. [54] explored the extraction of cefathiamidine from blood and zebrafish samples using the CPE-MAE. This application illustrates the versatility of the technique in bioanalytical contexts, overcoming challenges associated with complex biological matrices. The extraction of alkaloids and flavonoids from the Chinese leguminous plant (*Crotalaria sessiliflora*) using the CPE-MAE was efficiently performed by Triton X-100-NaCl-HCl in the assembly of phytochemical analysis of this botanical sample. Some pioneer studies, such as research conducted by Jia et al. [57], implemented this technique in human urine, successfully determining organophosphorus (OP) pesticides, including diazinon, quinalphos, fenthion, parathion-methyl, and phorate. Furthermore, Sikalos et al. [58] have focused on aqueous solutions of polycyclic aromatic hydrocarbons (PAHs), such as naphthalene, acenaphthylene, fluorine, anthracene, fluoranthene, and pyrene, demonstrating the efficiency of the CPE with the MAE as a pre-concentration step for subsequent gas chromatographic analysis. Zygoura et al. [59] explored the analysis of commercial plasticizers (diethylhexyladipate, and acetyltributylcitrate) in aqueous solutions after contact with PVC food packaging film, emphasizing the versatility of the CPE with the MAE in addressing complex matrices. These studies collectively highlight the efficacy of the CPE-MAE across varied sample matrices, showcasing its utility in environmental, biological, and industrial contexts for targeted analyte determination. Moreover, the described applications collectively emphasize the adaptability of the CPE-MAE in diverse analytical scenarios, ranging from environmental monitoring to pharmaceutical analysis and bioanalytics. The referenced studies provide valuable insights into the successful implementation of this technique, contributing to the advancement of sample preparation methodologies. On the other hand, no one determined the efficiency rate and limit of detection comparing the obtained extracts with some well-known and/or standardized analytic protocol.

### 5.2. The Cloud Point Extraction with Ultrasonic-Assisted Extraction (CPE-UAE)

The synergistic coupling of the CPE with the ultrasonic-assisted extraction (CPE-UAE) can also enhance extraction efficiency, selectivity, and adaptability across diverse sample matrices, establishing it as a powerful and transformative methodology for using the CPE at the industrial level. The CPE-UAE represents a cutting-edge approach that leverages the strengths of both cloud point extraction and ultrasonic-assisted extraction methodologies. When coupled with the CPE, ultrasonic-assisted extraction imparts additional impetus to the extraction process, optimizing the extraction parameters and improving overall performance. As for the CPE-MAE, this technique involves the acceleration power of an additional step in the extraction procedure to increase extraction efficiency (Figure 4). Using ultrasound power for better CPE results can contribute to an in-depth investigation of the fundamental principles of micelle-mediated extraction. Moreover, the CPE-UAE can reveal the complex interplay of phase separation dynamics within a micellar system and the cavitation-induced microstreaming and shockwaves generated by ultrasonic energy [10,13]. One of the most critical points during the CPE-UAE is the heating rate since temperature control before and during ultrasound irradiation is critical for preserving the integrity of thermolabile analytes. Precooling or preheating the sample before ultrasound irradiation can influence cavitation and extraction efficiency, offering an additional parameter to optimize based on the nature of the sample matrix. External cooling systems or feedback mechanisms aid in maintaining a controlled temperature, ensuring that the sample remains within the desired range [28].

Understanding the CPE-UAE principle is crucial for optimizing extraction conditions. This includes the optimization of the general parameters related to the CPE extraction, such as cloud point temperature, ultrasonic power, irradiation time, surfactant type, concentration, surfactant-to-sample ratio, and all others that are encompassed in Table 3. However, some specific operation parameters have to be potted for the CPE-UAE since the optimization of this technique needs to be a systematic approach considering the intricate interactions between surfactants, ultrasonic waves, and sample constituents. Table 4 highlights the operation parameters related to the UAE step in the whole CPE procedure for controlling temperature, adjusting surfactant concentrations, and fine-tuning ultrasonic parameters. Various ultrasound-related operation parameters have a specific role in shaping the advantages and disadvantages of this extraction technique. The optimization of ultrasound frequency, intensity, and irradiation time in the CPE coupled with the UAE requires a thorough understanding of the sample matrix and the physicochemical properties of the targeted analytes. Fine-tuning these parameters ensures efficient extraction while mitigating the risk of sample degradation or adverse effects. The synergistic benefits, such as accelerated mass transfer and improved selectivity, are balanced against challenges related to optimization complexities and compatibility issues, providing a realistic perspective.

Except for parameters shown in Table 4, some parameters, such as duty cycle, pulse mode, probe design, and geometry, as well as probe material, can contribute to the optimization of the extraction process. However, these parameters related to the ultrasound process have not been involved in any research investigation about the CPE-UAE until today. The duty cycle, representing the ratio of active ultrasound time to the total cycle time, is a crucial parameter influencing the overall energy delivered to the sample. Optimization involves a careful adjustment to balance efficient extraction with minimizing sample heating. Furthermore, operating in pulse mode, wherein ultrasound is applied intermittently, introduces rest periods between pulses. This mode aids in temperature control, which is particularly beneficial during longer extraction processes, preventing excessive heating and potential sample degradation. The material, design, movement, and geometry of the ultrasound probe are significant factors affecting the distribution of ultrasound energy and heat transfer in the sample. Tailoring probe selection to specific sample types or extraction requirements becomes essential for optimizing the extraction. One of the additional possibilities is utilizing horns in ultrasound devices, whose configuration and shape can play a role in affecting cavitation and energy distribution. Adjusting power density is essential to achieve uniform extraction across the entire sample. Additionally, the choice between low-frequency and high-frequency ultrasound modes introduces the adaptability of the UAE to the CPE extraction, with each mode having distinct effects on cavitation and extraction efficiency. The presence or absence of gases in the ultrasonic bath, as well as the immersion depth of the ultrasound horn, can influence cavitation efficiency. Introducing or controlling the gas atmosphere may impact extraction efficiency, especially when dealing with volatile analytes. These ultrasound-related parameters collectively contribute to the optimization of the CPE coupled with the UAE. However, the complex interplay of these parameters necessitates a systematic approach to achieve optimal results for specific analytical goals and sample characteristics.

The CPE-UAE finds applications across various analytical domains, including environmental, pharmaceutical, food safety, and bioanalysis. Case studies highlight its flexibility in handling complex sample matrices, showcasing its efficacy in extracting a wide range of analytes with enhanced sensitivity [10,13]. For example, the CPE-UAE for copper determination was investigated, and the obtained results revealed that under the optimized conditions, the limits of detection (LODs) for copper were 0.7 μg/L. Since the gained milestone represents better results than ultrasound-assisted liquid phase microextraction methods, Yang et al. [60] suggested using this technique for the determination of trace copper in real water samples with satisfactory analytical results. In the study by Temel and Gürkan [61], a method combining the CPE-UAE with spectrophotometry was developed for the extraction, preconcentration, and quantification of low levels of free formaldehyde from various beverage matrices. The proposed procedure employs a spectrophotometer operating at 538 nm, a widely available instrument in analytical research laboratories. This approach eliminates the necessity for an expert user, showcasing its user-friendly nature. The method demonstrates high sensitivity and selectivity, as well as excellent repeatability and reproducibility of the CPE-UAE, further contributing to its practical applicability. Similarly, Altunay et al. [62] proposed an indirect determination of the flavor enhancer maltol in foods and beverages using flame atomic absorption spectrometry after the CPE-UAE. The method involves the reduction of Cu(II) to Cu(I) by maltol at pH 6.5, followed by the selective interaction of Cu(I) with bathocuproine (BCP) in the presence of sodium dodecyl sulfate (SDS), resulting in the formation of a ternary complex. Under optimized conditions, the pre-concentration of a 35 mL sample solution enables the detection of maltol at a concentration as low as 1.24 mg/L. Biata et al. [63] reported a rapid and environmentally friendly approach using the CPE-UAE for efficient preconcentration and determination of antimony, tin, and thallium in food and water samples. The developed method was successfully applied to various samples, including certified reference materials, demonstrating its applicability for the rapid determination of the mentioned metals in diverse matrices. Motikar et al. [48] examined the CPE-UAE technique for the determination of polyphenols from pomegranate peel through the optimization of the CPE-UAE operation parameters. The maximum efficiency of extraction was obtained at 1:70 solid: solvent ratio, 8% (*v*/*v*) Triton X-114 at 55 °C, and pH 4.5 with 14% NaCl for 30 min. Obtaining phenols and flavonoids at rates of 96.28 mg GAE/g and 12.27 mg QE/g, respectively, this procedure can be defined as green and can be used in food and dietary applications. Enrichment of flavonoids from *Euonymus alatus* using PEG-400/water as an extractant was developed using the CPE-UAE. Using PEG-400 concentration of 16% (*w*/*w*), particle size of 80 mesh, solvent-to-material ratio of 60:1, extraction temperature of 90 °C and extraction time of 15 min, Mai et al. [64] obtained the extraction yields of catechin, dihydromyricetin and total flavonoids were 0.377–0.684 mg/g, 1.091–1.353 mg/g and 2.612–3.146 mg/g, respectively. Furthermore, a cost-effective method for iron determination in vegetable samples was developed using the CPE-UAE. The procedure involves simultaneous reagent extraction, complexation, and pre-concentration at 45 °C, utilizing an environmentally friendly natural chelating agent from *Dipterocarpus intricatus* dyer fruit in the presence of Triton X-114. The method demonstrated low limits of detection and quantification (0.03 and 0.09 mg·L−1, respectively), with precision values below 5%. Recovery ranged from 89.0 to 99.8%, and iron content in vegetable samples was determined as eco-friendly and convenient, presenting a reliable approach for iron content determination [65]. One more research study introduces a cost-effective and selective method for the determination of inorganic mercury in liquid matrices through the CPE-UAE extraction. The innovation lies in a modified procedure using amide copolymer through the extraction procedure, and spectrophotometric analysis after chelation with 2-aminobenzimidazole. The formed extraction procedure involved a 62.5-fold pre-concentration, with limits of detection and quantification of 0.24 and 0.22 μg/L, respectively. Precision ranges from 3.3% to 8.3%, meeting legislative requirements for mercury determination in water intended for human consumption. The method, validated through certified water analysis, is cost-effective and aligns with European legislation, demonstrating the potential for future analytical applications [66].

The reviewed literature and the actual research direct the significant potential of the CPE-MAE as well as the CPE-UAE, emphasizing the need for standardized methodologies, in-depth mechanistic studies, and the exploration of novel applications. It outlines potential advancements that could further enhance the efficacy and adoption of this integrated technique. The association of cloud point extraction with microwave or ultrasonic-assisted extraction techniques has emerged as a transformative force in analytical chemistry and extraction procedures. This synergistic integration not only enhances extraction efficiency but also opens new avenues for applications in complex sample matrices. As the scientific community continues to unravel the intricacies of these integrated methodologies, they are poised to redefine the landscape of green chemistry in the foreseeable future. The CPE-MAE and the CPE-UAE are both techniques employed in sample preparation, enhancing the efficiency of analyte extraction from complex matrices. In the comparison between the CPE-MAE and the CPE-UAE, the choice of energy source stands out as a crucial factor. The CPE-MAE relies on microwave energy, leading to rapid heating but potentially causing non-selective heating and degradation of heat-sensitive analytes. In contrast, the CPE-UAE employs ultrasonic waves, generating localized heating through cavitation. This selective and controlled energy application reduces the risk of analyte degradation, making the CPE-UAE more appealing for preserving the integrity of sensitive compounds. In the authors’ opinion, these are the main reasons for the deeper analysis and more optimization studies of the CPE-UAE compared with the same extraction, but using the MAE. Additionally, the CPE-UAE gains an advantage in accessibility, as ultrasonic equipment is more widely available compared to specialized microwave instruments. This makes the CPE-UAE accessible to a broader range of laboratories, including those with limited resources. Moreover, the CPE-UAE exhibits versatility across various sample types, handling solid, liquid, and semi-solid matrices effectively. Its effectiveness in complex matrices is particularly noteworthy, where ultrasonic waves can penetrate and disrupt sample structures, facilitating efficient extraction. Considering the environmental impact, the CPE-UAE tends to be perceived as more environmentally friendly. Ultrasonication generally requires less energy and produces fewer emissions than microwave heating. This aligns with the growing emphasis on green analytical methods in modern research and industry. Scalability and cost-effectiveness are additional factors favoring the CPE-UAE. Ultrasonic equipment is often more cost-effective and easily scalable for industrial applications, contributing to its practicality in large-scale operations. In summary, while both the CPE-MAE and the CPE-UAE have their merits, the wider accessibility, versatility, perceived safety, environmental friendliness, scalability, and cost-effectiveness of the CPE-UAE collectively position it as a more promising choice for future applications at the industrial level. This preference reflects the practical considerations and growing awareness of sustainable and efficient analytical methodologies within the scientific community and various industries.

### 5.3. Recent Examples of CPE-MAE and CPE-UAE for Recovering Bioactive Compounds from Different Plant Sources

Exploring the CPE-MAE and the CPE-UAE as a method for the recovery of bioactive compounds from plant sources with detailed insights is provided in the table. Interestingly, the diversity of plant materials and target bioactives, alongside the specific values of each influencing parameter such as surfactant type and concentration and operational conditions, are very varied and might be defined as unique combinations of plant material, target bioactives, surfactants, and extraction conditions, indicating a full image of the CPE’s adaptability and efficiency.

In the case of the CPE-MAE for recovering bioactive compounds from plant sources, only a few papers investigated this topic in the previous five years. This is additional proof that this technique surely requires more investigation and optimization, compared with the ultrasound-coupled extraction procedure, since researchers did not make a greater contribution in this field. As shown in Table 5, Campillo et al. [67] explored the CPE-MAE for recovery of vitamin K from vegetables (iceberg lettuce, romaine lettuce, lamb’s lettuce, escarole lettuce, kale, spinach, cress, turnip, parsnip, and carrot). A similar study was conducted by Yu et al. [68] in order to determine polyphenols and furocoumarins from fig leaves. Targeted bioactives are extracted using Triton X-45 and Triton X-114, respectively. The choice of surfactant and its concentration, such as 15% *w*/*v* for Triton X-45 and 8% *w*/*v* for Triton X-114, is customized to the nature of the bioactive compound. These cases use moderate temperatures, around 38 °C to 55 °C, and specific pH values to optimize extraction efficiency. The main difference between these two studies is in the different approaches in the case of the (no) addition of salt, as well as centrifugation parameter variability. The inclusion of salts like NaCl at varying concentrations is a common theme across these examples, assisting in the extraction process, but reaching an eco-friendly procedure requires minimizing the addition of any salts or additives in the extraction mixture. In more specialized cases, such as the extraction from pomegranate waste, Triton X-114 was used in a concentration of 8% *w*/*v* at a higher CPE temperature, longer extraction time, as well as salt concentration [48]. This variation suggests that the chemical nature of the target bioactive compounds, like polyphenols, significantly influences the choice of surfactants, operation parameters, and the CPE step repetition.

The use of the CPE-UAE in recovering antioxidants from plant sources has been explored in various studies, each adopting unique methodologies and targeting different compounds (Table 5). For example, the study by Altunay et al. [69], which focused on extracting metals like zinc, nickel, and cobalt from foods and vegetables, utilized Igepal CO-630 as a surfactant. The extraction conditions were mild, characterized by a low surfactant concentration and moderate temperature, suggesting suitability for sensitive compounds, although the extraction’s specificity and efficiency might be limited due to incomplete data on crucial parameters like pH and salt concentration. In contrast, Guo et al. [70] explored the extraction of polyphenols and alkaloids from mulberry using a higher concentration of Triton X-114, complemented by the addition of NaCl. This approach could potentially enhance the extraction of specific compounds, but the optimizing process is general and brings challenges in fully assessing the optimized conditions. A higher surfactant concentration and temperature were employed for the extraction of bioactives in the case of pomegranate waste [48] using an increased centrifugation speed. This more aggressive method, suitable for robust compounds, highlighted a different aspect of the versatility of the CPE-UAE procedure. Similarly, an investigation on the *Euonymus alatus* plant used an even higher concentration of PEG-400 for flavonoid extraction, with (NH_4_)_2_SO_4_ as a unique salting-out agent, potentially affecting the selectivity of the extraction [64].

One more plant source for obtaining antioxidants using the CPE-UAE technique is dandelion flowers [74]. In this study, the extraction of phenolic acids is targeted with Triton X-114 at a relatively high temperature. This set of parameters seemed customized for more temperature-stable compounds, though the lack of solid–liquid ratio information somewhat muddled the understanding of its efficiency. Temel et al. [71] investigated the extraction of vanadium (V) and vanadium (IV) in edible vegetal oils and vinegar using a significantly lower surfactant concentration and a lower temperature. This delicate approach suggested a focus on maintaining the stability of sensitive compounds and obtained good methodology for the determination of trace compounds.

Xu et al. [72] marked the possibility of using the CPE-UAE for the extraction of rutin and narcissoide from *Anoectochilus roxburghii* incorporating [C4mim] [PF6], an ionic liquid, with Triton X-114 for micelle-formation protocol. This combination indicated a potential enhancement in extraction efficiency for specific compounds. Meanwhile, the same surfactant, Triton X-114, is used for the extraction of iron from different green vegetables and offered a moderate approach but lacked detailed information on several parameters, hindering a full evaluation [65]. On the other hand, clingstone peach cannery waste as a potential source of polyphenols was used for the targeted extraction procedure using high values of Tween 80 concentration, temperature as well as long centrifugation time [45], indicating the robustness of the used methodology and extraction procedure for stable compounds. Lastly, Sun et al. [73] also targeted polyphenols but used pomegranate peel as a plant source and employed Triton X-110 at high concentrations and temperature, which indicated a possibly optimized method for higher yield or the extraction of specific polyphenols.

The use of additional techniques like the Microwave-Assisted Extraction (MAE) and the Ultrasound-Assisted Extraction (UAE) in the CPE protocol can enable further evolution of the CPE methodology. These techniques enhance the efficiency of the extraction process, indicating a trend toward more sophisticated methodologies in bioactive compound recovery, especially in the case of the UAE. Comparatively, the examples in Table 5 reveal both similarities and significant discrepancies in the CPE methodologies. A common thread is the use of surfactants and salts, which are central to the CPE process. However, the vast differences in surfactant types, concentrations, and extraction conditions like temperature, pH, and time reflect the highly specific nature of the extraction process for different bioactives and plant materials. This specificity emphasizes the complexity of bioactive compound extraction and the importance of customizing each aspect of the process to the target compound and plant source. Overall, this overview demonstrates the diverse approaches required in the extraction of bioactive compounds from plant materials. It also highlights the adaptability of the CPE methodology to different plant materials and bioactives, as well as the ongoing advancements in extraction technologies to improve efficiency and efficacy.

## 6. Conclusions and Future Perspectives

The cloud point extraction (CPE) is one of the most promising, environmentally friendly techniques for the recovery of antioxidants from food matrices. It is described as a rapid, efficient, precise, accurate, and convenient method that minimizes the use of toxic organic solvents. The CPE involves simple manual procedures, common laboratory supplies (glassware, pippets, and flasks), and equipment (heating plates and centrifuge). Therefore, there is no requirement for specialized equipment or extraction supplies. For the most part, cleanup of the extracted sample before chromatographic analysis is not required. The surfactants have low flammability and are relatively inexpensive. Moreover, quantitative yields can be acquired in a short time, and many samples may be processed simultaneously.

Nevertheless, some limitation factors of the CPE need to be considered and overcome in due time. Depending on the study, the utilized surfactants may result in analytical interferences and issues with detection limits, particularly if the analytes cannot be successfully isolated from the surfactants. In addition, extraction efficiencies decrease with increasing solute polarity and with highly volatile or thermally unstable compounds. Although the CPE is effective at a laboratory scale, scaling up the technique to industrial levels can be challenging. Large-scale CPE implementation requires careful consideration of variables such as food material volume, extraction time, and cost-effectiveness. These limitations provide opportunities for future enhancement and optimization.

Based on the reviewed articles over the last six years (2018–2023), it can be stated that even though there are still a lot of works using the traditional CPE procedure, recently published works attempted to improve the CPE using innovative approaches such as the ultrasound-assisted CPE or the microwave-assisted CPE. Novel approaches aim to improve certain parameters of the CPE methodology. This involves increasing extraction efficiency, lowering the CPT, decreasing the amount of utilized reagents, accelerating the extraction step, and improving the removal of potential interferents from analyzed matrices, with economic benefits. Additionally, exploring and evaluating new surfactants or biosurfactants to recover food-bioactive compounds is required. At the inception of the CPE, the univariate optimization approach was employed in most published papers, while nowadays, multivariate optimization techniques like response surface methodology, central composite design, and Box–Behnken experimental design are becoming more and more common. Future research can focus on the reduction in experiment numbers, which means saving time, energy, and chemicals. The advancement of automation, microfluidic systems, and online system connectivity presents challenges for the future as well. Exploring the extraction of other bioactive compounds, such as proteins and enzymes, as well as the simultaneous extraction of multiple bioactive compounds by the CPE, holds promise and can provide valuable insights for the scientific community.

## Figures and Tables

**Table 1:** The four categories of surfactants utilized in CPE [12].

Category	Surfactant Examples	Properties
Non-ionic	Polyoxyethylenes (Genapol X-080, Triton X-100, Triton X-114, Tween 80)	Uncharged hydrophilic head
Anionic	Sodium dodecyl sulfate, ammonium lauryl sulfate, sodium laureth sulfate	The hydrophilic group contains an anionic moiety, such as carboxylate, sulfonate, or sulfate
Cationic	Cetyl trimethylammonium bromide, methylbenzethonium, benzalkoniu	The hydrophilic head contains positive groups, such as quaternary ammonium
Zwiter anionic	4-(Dodecyldimethyl ammonium) butyrate, erucyl amidopropyl betaine	Cationic, anionic, or neutral, depending on the solution’s pH

**Table 2:** Overview of CPE for recovering antioxidants from plant sources [16,34,35,36,37,38,39,40,41,42,43,44,45,46,47,48,49].

Plant Material	Target Group of Antioxidants	Surfactant Type	Surfactant Concentration	Temperature (°C)	pH	Time (min)	Solid–Liquid Ratio	Salt	Salt Concentration (% *w*/*v*)	Centrifugation Speed (rpm)	Centrifugation Time (min)	CPE Step	Ref.
Camu camu residue	Polyphenols	Triton X-114	7% *w*/*v*	30	3.2	180	-	NaCl	6	-	-	1	[36]
Red grape pomace		Brij S20 and Poloxamer 407	3% *w*/*v*	25	4	45	1:10 *w*/*v*	-	-	3500	20		[37]
*Acalypha fruticosa* powder		Tween 20	8 mM	70–80	-	30	1:100 *w*/*v*	KCl	2	4000	10		[38]
*Carica papaya* leaves		Pluronic L-61	10% *w*/*w*	40	-	10	0.1% (*w*/*w*)	-	-	10,000			[39]
Apricot cannery wastewater		Peg 8000	2% *w*/*v*	65	3.5	20	-	NaCl	3	3500	5	2	[40]
Wine sludge waste		Lecithin	5% *w*/*v*	40	3	30	-	NaCl	5	3500	15	3	[41]
Olive mill wastewater			12.5% *w*/*v*		3.5				10	4500	5		[42]
			3% *w*/*w*						30				[46]
Olive process wastewater		Tween 80	10% *w*/*v*	70	2	30	-	-	-	-	-	1	[43]
Peach waste streams			5% *w*/*v*	65	3.5	20		NaCl	3	3500	5	2	[45]
Unripe and ripe peaches				45	2.5				6				[46]
Pomegranate peel	Polyphenols, flavonoids	Triton X-114	8.22% *w*/*v*	36.80	4	30	0.5 g/50 mL	NaCl	4	8000	10	1	[47]
			8% *w*/*v*	55	4.5		1:30 *w*/*v*		14	12,000			[48]
Tomato wastewater	Carotenoids	Lecithin	1 or 2% *w*/*v*	45	3.5	20	-	NaCl	35.6	4500	5	3 or 2	[49]
Brown microalgae		Tween 20	0.046 mol/L	25	-	140	0.02 mg/mL	-	-	5000	40	1	[50]
Green microalgae	Chlorophylls	C26H56ClP	250 mM	25	-	30	0.01 g/mL	-	-	5000	30	1	[51]
Spinach leaves		C11-C13 9EO’s	12.4 mM	41	-	30	0.07 *w*/*w*	-	-	-	-	1	[18]

**Table 3:** A wide-ranging list of operation parameters for the CPE coupled with MAE [15,26].

Operation Parameters	Parameter Arrays	Short Explanation
Cloud Point Temperature	Process	The temperature at which phase separation occurs is a critical parameter. Optimization ensures that the CPT is conducive to the efficient extraction of the target analytes.
Temperature		The temperature during microwave irradiation should be controlled to avoid degradation of analytes and to optimize the phase separation process.
Stirring or Agitation		Stirring or agitation of the sample during extraction can enhance mass transfer and improve efficiency.
Microwave Power and Irradiation Time		Microwave power and irradiation time directly influence the heating and extraction efficiency. Optimization prevents sample degradation and achieves maximal extraction yields.
Instrumentation Parameters		Specific parameters of the microwave instrument, such as frequency and mode of irradiation, need to be optimized for compatibility with the CPE.
pH of the Extraction Medium		The pH of the extraction medium affects the solubility of analytes and the stability of micelles. Optimal pH conditions should be established for efficient extraction.
Sample Pretreatment	Sample	Preparing the sample through appropriate pretreatment methods, such as grinding or homogenization, can impact the accessibility of analytes during extraction.
Sample Matrix Characteristics		The nature of the sample matrix, including its complexity, viscosity, and potential interference, must be considered for the effective CPE-MAE
Sample Size		The amount of sample used can impact the extraction efficiency. Optimization involves determining the optimal sample size for the given system.
Surfactant Type and Concentration	Surfactant	The choice of surfactant significantly affects the CPE. Selection based on its critical micelle concentration and compatibility with microwave irradiation is crucial.
Co-Surfactant/Additives		The addition of co-surfactants or other additives may enhance the solubilization of certain analytes or improve phase separation, contributing to overall extraction efficiency.
Surfactant-to-Sample Ratio		The ratio of surfactant to the sample is critical for achieving phase separation and maximizing the concentration of analytes in the surfactant-rich phase.
Nature of Analytes	Analytes	The physicochemical properties of the target analytes, such as solubility and volatility, influence their extraction behavior. Understanding these properties is crucial for optimizing extraction conditions.
Extraction Solvent and Volume		The choice of extraction solvent, its compatibility with surfactants, and the volume used influence the extraction efficiency. Optimization ensures an appropriate solvent for the target analytes.
Microwave Vessel Material	Safety	The choice of vessel material for microwave irradiation can influence the heating efficiency and should be considered during optimization.
Safety Precautions		Ensuring proper safety measures during microwave irradiation is crucial to prevent accidents and ensure the integrity of the extraction process.

**Table 4:** A list of ultrasound-related operation parameters that influence the CPE-UAE [15,26].

Ultrasound-Related Parameters	Short Explanation
Ultrasound Frequency	Higher frequencies are associated with smaller cavitation bubbles but may have limited penetration. Lower frequencies penetrate deeper but may result in larger bubbles. Optimization involves selecting a frequency that balances efficient cavitation and penetration based on the nature of the sample matrix and desired analyte extraction.
Ultrasound Intensity	It influences cavitation effects and heating during extraction. Optimization involves determining the level that promotes effective cavitation without causing excessive sample heating or degradation.
Duration of Ultrasound Exposure	The duration directly influences the efficiency of analyte release from the matrix. Optimization of irradiation time involves finding the balance between sufficient extraction and minimizing sample degradation. Shorter irradiation times may not fully exploit cavitation effects, while excessively long times may lead to undesired effects.
Cavitation rate	Cavitation is the formation, growth, and collapse of bubbles in a liquid medium. It creates localized microenvironments with high temperatures and pressures, facilitating the release of analytes from the sample matrix. Longer irradiation times may enhance cavitation effects, but careful control is necessary to avoid excessive heating, which can degrade sensitive analytes.

**Table 5:** Overview of CPE for recovering antioxidants from plant sources [48].

Plant Material	Target Bioactives	Surfactant Type	Surfactant Concentration	Temperature (°C)	pH	Time (min)	Solid–Liquid Ratio	Salt	Salt Concentration (% *w*/*v*)	Centrifugation Speed (rpm)	Centrifugation Time (min)	CPE Step	Ref.
CPE-MAE													
Vegetables *	Vitamin K	Triton X-45	15% *w*/*v*	38	7	10–20	nd	NaCl	0.04	3500	3	1	[67]
fig (*Ficus carica* L.) leaves	*Polyphenols* and *furanocoumarins*	PEG8000	2.5% *w*/*v*	40	nd	10.27	19.95 mL/g	/	/	12,000	10	1	[68]
*Pomegranate peels*	Polyphenols	Triton X-114	8% *w*/*v*	55	4.5	30	1:70	NaCl	14	12,000	10	2–3	[48]
CPE-UAE													
foods and vegetables **	zinc, nickel and cobalt	Igepal CO-630	0.2% *w*/*v*	50	5	10	nd	nd	nd	4000	5	1	[69]
*Mulberry* leaves	*polyphenols* and *alkaloids*	Triton X-114	3% *w*/*w*	nd	nd	nd	1:35	NaCl	0.05 M	3800	5	1	[70]
*Euonymus alatus*	*flavonoids*	PEG-400	16% *w*/*w*	55	3.5	15	1:60	(NH4)_2_SO_4_	6.7	4000	5	2	[64]
edible vegetal oils and vinegar	Vanadium types (V) and (IV)	Triton X-114	0.001–0.01074% *w*/*v*	40	4	5	nd	NaNO_3_	0.15 mol/L	4000	10	1	[71]
*Anoectochilus roxburghii* (Wall.) Lindl.	rutin and narcissoside	20% [C4 mim] [PF6] and Triton X-114	[C4 mim] [PF6]:Triton X-114 = 2:23	45	3	10	1:60	NaCl	0.25 g/mL	4000	10	1	[72]
Green vegetables ***	iron	Triton X-114	0.3% *w*/*w*	45	5.5	nd	nd	/	/	5000	10	nd	[65]
Clingstone Peach Canneries waste	polyphenols	Tween 80	10% *w*/*w*	65	3.5	20		NaCl	3	4500	20	2	[45]
Pomegranate peel		Triton X-110	10% *w*/*w*	70	4	40	1:40	NaCl	14	6000	20	1	[73]
		Triton X-114	8% *w*/*v*	55	4.5	30	1:70		14	12,000	10	2–3	[48]
Dandelion			5% *w*/*v*	60	3.5		nd		10	6000	5	1	[74]

* iceberg lettuce, romaine lettuce, lamb’s lettuce, escarole lettuce, kale, spinach, cress, turnip, parsnip, and carrot ** Lettuce, spinach, mushroom, green pepper, tomato, broccoli, mint, and peas, as well as green lentils, red lentils, peanuts, nuts, oats, rice, and almonds; *** Chinese kale, sweet basil, Tiliacora triandra leaf, Siamese neem flower, wild betel leaf bush, Thai copper pod, peppermint leaf, and Turkey berry fruit.

## Data Availability

All the data is contained within the article.
